# Household Food Insecurity: Comparison between Families with and without Members with Disabilities

**DOI:** 10.3390/ijerph17176149

**Published:** 2020-08-24

**Authors:** Jong Eun Park, So Young Kim, Se Hee Kim, Eun Ju Jeoung, Jong Hyock Park

**Affiliations:** 1 Department of Medicine, College of Medicine, Chungbuk National University, Cheongju 28644, Korea; je.park0525@gmail.com; 2 Department of Public Health and Preventive Medicine, Chungbuk National University Hospital, Cheongju 28644, Korea; 3 College of Medicine/Graduate School of Health Science Business Convergence, Chungbuk National University, Cheongju 28644, Korea; 4 Harvard T. H. Chan School of Public Health, Harvard University, Boston, MA 02115, USA; 5 Department of Food and Nutrition, Chungbuk National University, Cheongju 28644, Korea; sh02973@naver.com (S.H.K.); dmswn5506@naver.com (E.J.J.)

**Keywords:** household food insecurity, disability, disability type, Korea National Health and Nutrition Examination Survey, Household Food Security Survey Module

## Abstract

Although the high rate of food insecurity among people with disabilities and their households has emerged as an important concern in public health and nutrition policy, the available data on these issues are still too limited to fully understand this phenomenon. This study aimed to compare the prevalence of food insecurity between households with and without persons with disabilities and to explore which sociodemographic and disability characteristics are associated with household food insecurity among households with members with disabilities. The data of 2690 households with and without members with disabilities from the 2013 Korea National Health and Nutrition Examination Survey were analyzed. Household food insecurity was more prevalent among households including persons with disabilities than among those without such members. The likelihood of experiencing food insecurity was especially high in households having a female head with a disability (odds ratio (OR) = 1.98); working-age adults with disabilities (OR = 1.70); members with disabilities who were not economically active (OR = 1.53); and members with mental disabilities (OR = 2.81), disabilities involving internal organs (OR = 4.38), or severe (grades 1–3) disabilities (OR = 1.73). The findings indicate that the disability status and sociodemographic characteristics of disabled family members are closely associated with household food security status.

## 1. Introduction

Food insecurity is a major health and nutrition challenge and remains high on the policy agendas of most countries [1,2]. There have been many efforts towards defining and improving food insecurity at the global, national, household, and individual levels, and substantial progress has been made to this end over the last few decades [1,2,3]. Nevertheless, approximately 2 billion people worldwide (an estimated 26.4% of the world population) experienced food insecurity in 2018 [4]; thus, a vast number of people still do not have reliable access to sufficient safe and nutritious food. 

The Food and Agriculture Organization of the United Nations (FAO) [5] has defined food security as “a situation that exists when all people, at all times, have physical, social, and economic access to sufficient, safe, and nutritious food that meets their dietary needs and food preferences for an active and healthy life.” Household food insecurity (HFI) applies this concept at the family level [6]. 

Although limited availability of adequate foods can occur for various reasons, many food security measures concerned with the individual and household levels, including the Household Food Security Survey Module (HFSSM) of the United States Department of Agriculture’s (USDA), focus on HFI arising specifically from a lack of financial resources to obtain sufficient food [7]. Indeed, many studies on the determinants of food insecurity have shown that low socioeconomic status, indexed by unemployment, low income, and a low education level [7,8,9,10], as well as certain demographic characteristics (e.g., living alone, single-parent households with children, and households headed by women) were associated with food insecurity [7,8,9,10]. An important and obvious fact is that food insecurity is primarily related to economic resources and is more prevalent among disadvantaged and vulnerable groups.

Current literature on the social, economic, and health conditions of persons with disabilities (PWDs) suggests that this may be the most vulnerable group in society [11,12,13]. PWDs are faced with challenges and difficulties in many area of their lives, including access to legal and social services, social opportunities, health, education, employment, and personal development [13]. Moreover, PWDs are more likely to experience food insecurity [14,15].

Several studies reported that the prevalence of HFI among households with PWDs was well above that for the general population [14,15,16,17]. Similarly, food-insecure households were more likely than food-secure households to include someone with a disability [9,18]. Households with PWDs are more likely to face unemployment, reduced earnings, and significant additional expenses resulting from their disability [19,20,21]. Moreover, the health of individuals with disabilities is often compromised by food insecurity [22], and the negative health consequences of insufficient food or a low-quality diet (e.g., overweight/obesity, and physical and mental health problems) may be greater for PWDs [23,24,25,26,27]. 

However, despite food insecurity being a particular concern among PWDs, data on the food security status of households with PWDs in most countries, except in some high-income Western countries, remain insufficient [15,16,17]. Furthermore, the underlying mechanisms by which disabilities affect food security are not fully understood [17] because very few attempts have been made to understand the social, economic, and health disparities experienced by PWDs; even survey data addressing their food and nutrition intake are lacking. Furthermore, in Korea, there has also been little recognition or discussion to improve the unsatisfactory food security status or nutritional conditions of PWDs.

To fully understand and address the issues associated with disparities in access to sufficient and nutritionally adequate food, it is necessary to expand the focus of food insecurity studies to include populations with disabilities. To provide scientific evidence to further publicize and to discuss the issue of food insecurity in PWD and households with PWD in Korea, this study compares the food security status between households with and without PWD using data in the 2013 Korea National Health and Nutrition Examination Survey (KNHANES). Furthermore, we explore whether particular sociodemographic and disability-related characteristics of PWD are associated with HFI.

## 2. Materials and Methods 

### 2.1. Study Population

This study used data acquired during the first year (2013) of the 6th KNHANES, conducted by the Korea Centers for Disease Control and Prevention (KCDC) between 2013 and 2015. Only data from 2013 were used because data related to disability status were obtained only during the first year of the 6th KNHANES. 

The KNHANES was designed to obtain information on the health and nutritional status of a nationally representative sample of Koreans through health examinations and extensive interviews. Additional details on the design of the survey are presented elsewhere [28]. 

Among 3182 households (8018 individuals) with at least one household member aged 1–96 years, only the 3161 households that reported the disability status of family members were recruited to this study. Households that did not respond to the household food security survey or did not provide information about the head of household were excluded. The final sample included 2690 households that provided complete information on disability and food security status. In total, 384 households included PWD, while 2306 households had no such members. 

All participants provided written informed consent, and the institutional review board (IRB) of the KCDC approved the study (approval number: 2013–07CON–03–4C).

### 2.2. Disability-Related Characteristics

Self-reported information on disability registration status, type, and severity was obtained from each individual. Korea has a national registration and grading system for PWD based on the Act on the Welfare of Persons with Disabilities [29]. The registry includes 15 legally defined types of disability. In the present study, disability types were reclassified as follows: (1) physical disability (physical impairment, brain lesion, or facial disfigurement); (2) visual disability; (3) auditory or linguistic disability; (4) mental disability (intellectual or psychological disorders, or autism); and (5) internal organ disability (kidney, cardiac, respiratory or liver dysfunction; intestinal/urinary fistula; or epilepsy). 

The severity of each disability type was scored from 1 to 6. We divided the disabilities into severe (grades 1–3) and mild (grades 4–6) categories. When households had two or more members with disabilities, we analyzed the data of the more prominent household member (head of household or working-age adult). 

We also examined the relationships of the PWD to their families (e.g., head of household, spouse, son/daughter, or other relative) and recorded whether they were working-age adults (aged 19–64 years) and whether they were economically active.

### 2.3. Assessment of Household Food Insecurity

Information on household food situation was collected from the member who prepared meals for their families in each household. First, all individuals were asked whether they had participated in food assistance programs during the last 12 months. The following question was then asked to explore experiences of food insufficiency at the household level: “Which of the following best describes the food situation in your household in the last 12 months: (1) enough of the kinds of food we want to eat; (2) enough but not always the kinds of food we want; (3) sometimes not enough to eat; (4) often not enough to eat?” We classified households as food-insufficient if they indicated that they sometimes or often did not have enough food. 

Food security measures for Korean National Health and Nutrition Examination Survey was developed based on the US-HFSSM.

HFI was measured using food security measures for the Korean population developed based on the USDA’s 18-item HFSSM [30,31]. This measure consists of a series of questions about the conditions and behaviors that characterize households having difficulty meeting basic food needs, including three questions about the food conditions of the household as a whole and seven questions about the food conditions of adults specifically. For households with children, eight additional questions about food conditions were included [7].

Based on their questionnaire data, the food security status of each household was categorized as food-secure (raw score 0–2), mildly food-insecure without hunger (raw score 3–5 for households without children; raw score 3–7 for households with children), moderately food-insecure with hunger (raw score 6–8 for households without children; raw score 8–12 for households with children), or severely food-insecure with hunger (raw score 9–10 for households without children; raw score 13–18 for households with children). The mildly food-insecure group (7.7% of the sample), moderately food-insecure group (1.6%), and severely food-insecure group (0.2%) were combined, and HFI status was then classified as food-secure or food-insecure.

### 2.4. Other Variables

To understand the sociodemographic characteristics of the households with and without PWDs, the gender, age, marital status, educational level, and occupation type of the head of the household were obtained in addition to household size (number of household members), family type, presence of children aged ≤18 years, number of economically active household members, household income (quartiles), and place of residence.

### 2.5. Statistical Analyses

Differences between households with and without PWD were assessed using the chi-square (χ^2^) test for categorical variables and Student’s *t*-test for continuous variables. The prevalence of HFI was estimated based on the characteristics of the head of the household, the household as a whole, and disability status.

We used multivariate logistic regression to assess the associations between household members’ disability status and HFI and obtained odds ratios (ORs) and 95% confidence intervals (CIs) for HFI. Covariates were included in the multivariate models. Model 1 included the demographic characteristics of the head of the household and the household itself, as listed above; model 2 included the covariates in model 1 plus the educational level of the household head and the number of economically active household members; and model 3 included the covariates in model 2 plus household income.

All statistical analyses were performed using the SAS statistical software (SAS Institute Inc., Cary, NC, USA). Statistical significance was set at *p* < 0.05.

## 3. Results

### 3.1. Characteristics of Households with and without Persons with Disabilities

As shown in Table 1, the characteristics differed significantly between the households with and without PWDs. Heads of households with PWDs were more likely to be female (*p* = 0.02), older (*p* < 0.001), and unemployed (*p* < 0.001) and to have less education (*p* < 0.001) compared to those of households without PWDs. 

Households with PWDs tended to have fewer family members (*p* = 0.003), no children aged 18 years or younger (*p* < 0.001), and fewer economically active members (*p* < 0.001) compared to households without such members. Of the households with PWDs, 40.1% were in the lowest household income category (first quartile) compared to only 23.2% of households without a PWD (*p* < 0.001).

Of the households with at least one member with a disability, 7.3% had two or more members with disabilities, 51.8% had a working-age adult with disabilities, and 37.5% had members with disabilities who were economically active. Most households with PWDs had at least one key household member with a disability, such as the head of household or their spouse. The majority of PWDs had physical disabilities, followed by auditory or linguistic, visual, mental, and internal organ disabilities; most had mild disabilities (grades 4–6). 

### 3.2. Food Access and Food Security Status 

The findings regarding household food situation are presented in Table 2. Of households that included PWDs, 13.3% benefitted from food assistance programs compared to 6.4% of households without such members (*p* < 0.001). Representatives of households were asked, “Which statements best describes the food situation in your household in the last 12 months?” Among households with PWD, 10.4% responded that they “sometimes or often did not have enough food to eat” (*p* = 0.001).

Households with PWDs were also more likely to experience some kind of food insecurity than those without such members. Mild food insecurity was observed in 11.5% of households with PWDs and in 7.0% of households without PWDs. Moderate or severe food insecurity was observed in 2.6% of households with PWDs and in 1.6% of households without PWDs (*p* value = 0.004). In addition, households with PWDs were more likely than those without to report insecurity for every indicator of the HFI. More than one-quarter (26.0%) of households with PWD reported having worried that their food would run out before they obtained money to buy more. About 25.3% reported that they could not afford to eat balanced meals; 10.9% reported that the food they bought did not last and that they did not have money to get more (all *p* < 0.05). 

### 3.3. Prevalence of Household Food Insecurity

**Figure 1:**
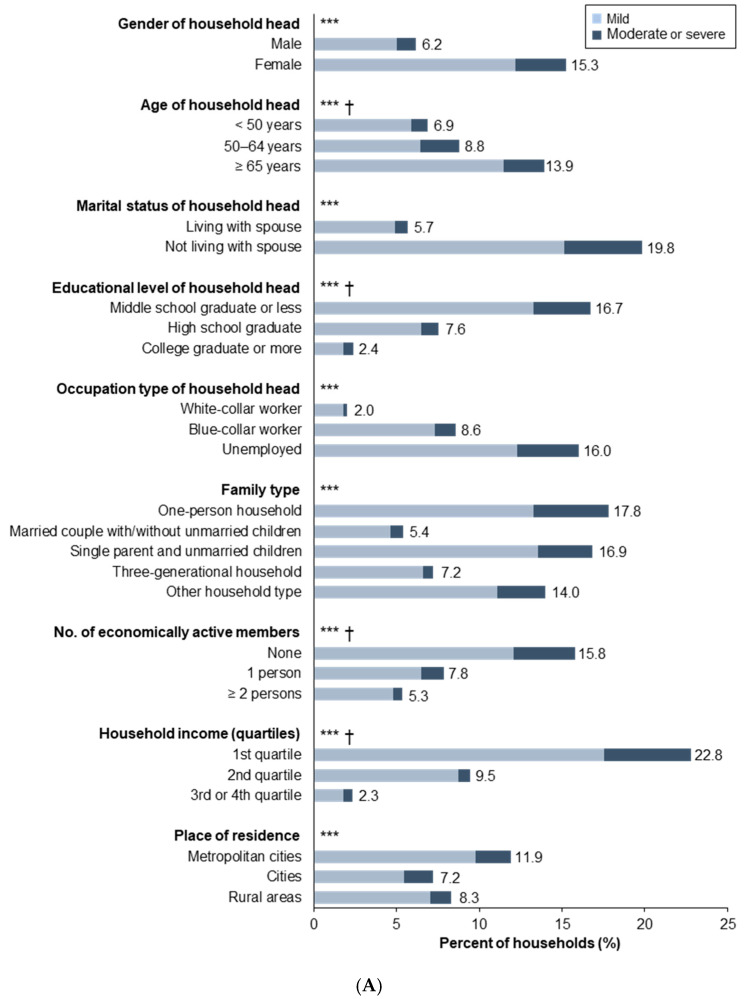

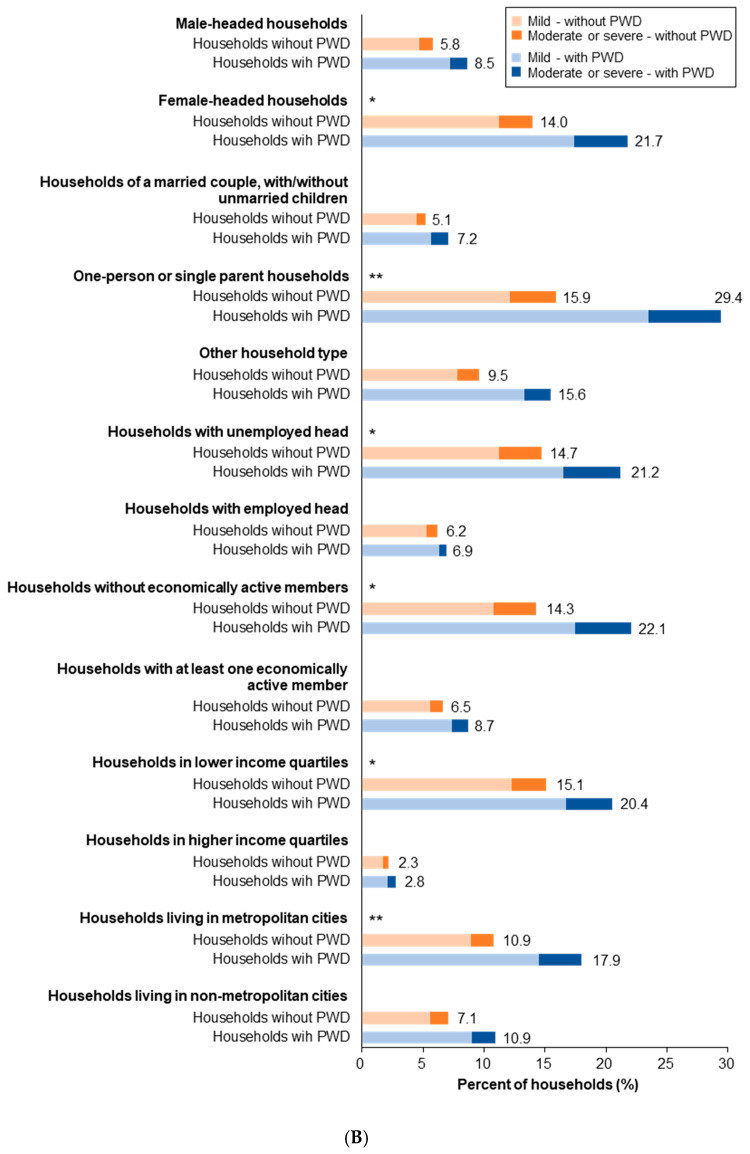

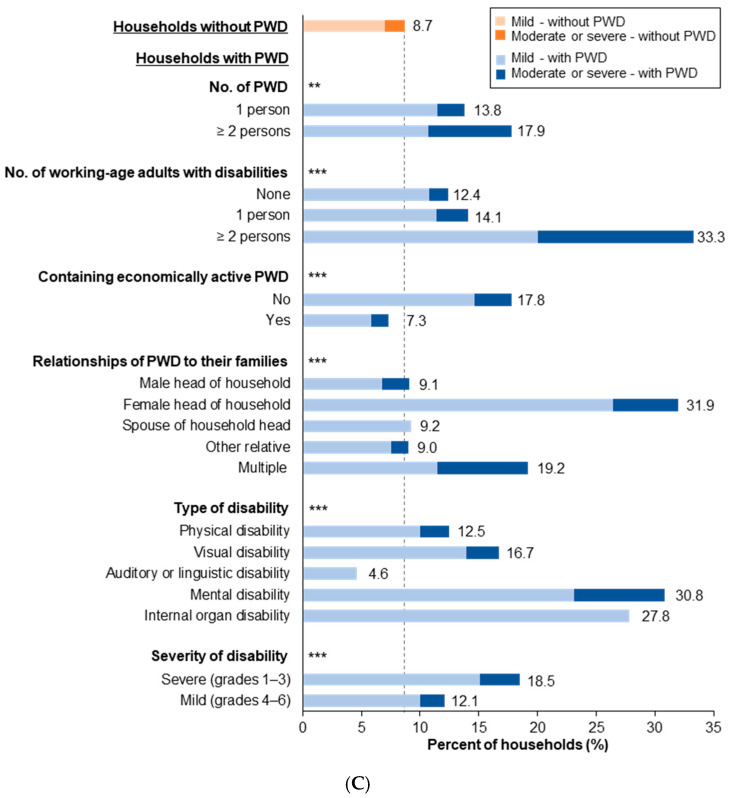
The prevalence of HFI according to sociodemographic characteristics and disability status: The values in panels **A** (Prevalence of household food insecurity (HFI) according to the selected characteristics of the household head and household), **B** (Comparison of HFI between households with and without persons with disabilities according to different sociodemographic characteristics.), and **C** (Comparison of food insecurity between households with and without persons with disabilities according to disability-related characteristics.) represent the rate of HFI, including mild, moderate, and severe food insecurity. The asterisk (*) in panel A indicates a statistically significant difference among the sociodemographic groups among the entire population, and the asterisks in panels B and C indicate a statistically significant difference between among households with and without PWDs (* *p* < 0.05; ** *p* < 0.01; and *** *p* < 0.001). The obelisk (†) in panel A represents a statistically significant trend for ordinal variables (*p* < 0.001). Abbreviations: HFI, household food insecurity; PWD, person with disabilities.

HFI, including mild to severe food insecurity, was more common among families headed by women (15.3% of households), the elderly (13.9%), and those without spouses (19.8%) (Figure 1A). One-person households (17.8%), single-parent households (16.9%), and households with unemployed heads (16.0%) or living in metropolitan cities (11.9%) were also more vulnerable to HFI. In addition, the rate of HFI increased dramatically with a decrease in the household head’s educational level, household income, and number of economically active household members (all *p* for trend < 0.001).

Households with PWDs were more likely to experience HFI than households without such members, especially in households headed by women, one-person or single parent households, households with no employed head or economically active members, households in the lower income quartile, and households living in metropolitan cities (Figure 1B).

Of the households including two or more members with disabilities, 17.9% experienced compromised food security compared to 13.8% of those with only one such member and 8.7% of those without PWDs (Figure 1C). Furthermore, we found that HFI was more prevalent among households with PWDs when the household included more working-age adults with disabilities (33.3%). In addition, among households with PWDs, the rate of HFI was significantly higher if the PWD was economically inactive (17.8%); was a female head of household (31.9%); or had a mental (30.8%), internal organ (26.3%), or severe (grades 1–3) (18.5%) disability.

### 3.4. Association between Disability Status of Household Members and Household Food Insecurity

In model 1, relative to households without PWDs, the likelihood of HFI was 64% higher in households with PWDs after adjusting for the demographic characteristics of the household head and the household itself (OR = 1.64, 95% CI = 1.16–2.31) (Table 3). Moreover, as the number of PWD increased, the likelihood of experiencing HFI increased by 51% (OR = 1.51, 95% CI = 1.13–2.02). However, these associations lost significance after additional adjustment for socioeconomic variables, such as educational level of household head, number of economically active household members, and household income. 

A higher likelihood of HFI was also observed in female-headed households that included PWD (OR = 2.11, 95% CI = 1.37–3.25) and households having a female head with a disability (OR = 2.37, 95% CI = 1.40–4.02). After further adjustment for household income in addition to covariates in model 2, the associations were greatly attenuated but still significant, with ORs of 1.63 (95% CI = 1.04–2.55) and 1.98 (95% CI = 1.14–3.43), respectively.

After adjusting for all demographic and socioeconomic variables, having working-age adults with disabilities in the household was independently associated with the likelihood of HFI, with an OR of 1.70 (95% CI = 1.07–2.70). Households with PWDs who were economically inactive increased the likelihood of HFI by 98% (95% CI = 1.35–2.91), but the association was attenuated after further adjustment for socioeconomic variables, with an OR of 1.53 (95% CI = 1.01–2.31). 

Additionally, compared to households with no PWDs, those with persons having a mental, internal organ, or severe (grades 1–3) disability had 4.19- (95% CI = 1.69–10.36), 4.65- (95% CI = 1.54–14.09), and 2.13-fold (95% CI = 1.27–3.56) higher odds of HFI, respectively, in model 1. After additional adjustment for educational level of household head, number of economically active household members, and household income, these associations were attenuated but still remained statistically significant: the odds of HFI were 2.81 (95% CI = 1.08–7.28) for mental disability, 4.38 (95% CI = 1.21–15.79) for internal organ disability, and 1.73 (95% CI = 1.01–2.99) for severe (grades 1–3) disability. 

## 4. Discussion

Although previous studies have assessed food insecurity according to geographic and sociodemographic characteristics, few have compared HFI between households with and without PWDs or identified factors associated with HFI in households with PWDs [15,16,17]. To the best of our knowledge, this examination is the first of its kind to be conducted in a representative sample of Koreans. 

We found that the sociodemographic characteristics of households with PWDs differed considerably from those of households without such members. For example, households with PWDs were more likely to have low socioeconomic status and a female head of household, where both of these factors are known to be related to poverty and food insecurity. Based on our a priori understanding of the basic sociodemographic characteristics of households with PWDs, we focused on certain sociodemographic characteristics and types of disability in our investigation of HFI, i.e., on those that represent the difficulties confronting individuals and households with disabilities.

The descriptive statistics and multilevel logistic regression models consistently showed that HFI was more common among households with PWDs than among households without such members. In particular, households with PWDs were more vulnerable to HFI when the head of household was a woman; when an adult member of working-age had a disability; when a household member with a disability was a female head of household or was not economically active; and when the household included a member with a mental, internal organ, or severe (grades 1–3) disability. These results are more robust than those of previous studies on disability and food insecurity, as most remained significant after controlling for a notable potential confounder, namely household income.

However, as more socioeconomic variables were added to the logistic regression model, the odds ratio of HFI was further attenuated and was no longer statistically significant in some subgroups. For all households with PWD, a higher likelihood of HFI was no longer statistically significant after additional adjustment for socioeconomic variables that represent the economic status of the household. Moreover, the associations between households with members with disabilities and with food insecurity were not observed in the less vulnerable subgroups in terms of economic and disability severity. For example, this included instances when the head of the household that included the PWD was male; when the household member with a disability was a member other than the household head, was of a non-working age group, or was economically active; and when a household included a mild (grades 4–6) disability.

Disability has been consistently related to an increase in HFI in previous studies [9,15,17,20]. Stronger associations between disability and HFI have generally been observed for young and middle-aged adults with disabilities compared to for older adults or children with disabilities [15], and female disability has been shown to have a greater effect on HFI than male disability [32,33]. Work-limiting disabilities, functional limitations, mental disabilities (including cognitive or developmental disabilities as well as mental health conditions) [15,17,20,21,34], and multiple and severe disabilities [34,35] were also shown to be particularly detrimental to HFI compared to other forms of disability. Furthermore, these previous findings are largely consistent with our results despite the differences in populations and study designs.

Some studies have focused on the disability status of certain household members, such as household heads or working-age adults, in relation to the economic status of the overall household. HFI was more prevalent among households in which such members had disabilities [21,32,34,35]. For example, 33.5% of households with working-age adults who had disabilities that rendered them unable to work were food-insecure compared to 24.8% of households in which the disability did not hinder work and 12.0% when there was no disability [21]. Households headed by PWD were also at higher risk of HFI compared to those headed by nondisabled persons (20% and 13%, respectively) [32]. In fact, households headed by PWD had significantly lower household income-to-needs ratios than their counterparts without PWD [32].

Several factors may explain the effects of disability on HFI. First, individuals and households affected by disabilities are more likely to have fewer economic resources due to limited opportunities for education, skills development, and employment, all of which result in reduced earnings: These limitations can also lead to fewer employment opportunities and reduced earnings for other household members who need to care for the member with a disability as well as for PWDs themselves [21,32,36,37]. In fact, on average, PWDs are two to six times less likely to be employed [38] and households with PWDs may experience a sharp decline in their household income, especially when income-earning household members, such as the heads of households and working-age adults, have disabilities [21,32,37]. Higher additional expenditures among PWD, including on medical care, adaptive equipment and assistive technology (e.g., wheelchairs, hearing aids, and prostheses), and other disability-related expenses, may represent another explanation [19,32,39,40]. Additional expenditures related to managing a disability may force households with PWDs to make trade-offs between basic needs (such as food) and disability-related expenses [32,39]. In addition, several other reasons, such as various barriers (e.g., transportation) to accessing food supplies or PWD’s coping strategies for food hardship, may partially explain the unique effect of disability on food insecurity [32].

In this study, HFI was primarily seen when the member with a disability was a female head of household or had a particular type of disability such as a mental, internal organ, or severe (grades 1–3) disability. This may be because these groups face more difficulties in entering the labor market, are at risk of incurring significant expenses resulting from their disability, and have relatively poor strategies to cope with these difficulties [32,41,42,43]. Indeed, the probability of experiencing HFI was observed to increase as out-of-pocket medical expenditure increased [40]. This suggests that the higher probability of HFI among households with persons with internal organ disabilities is largely attributable to the higher financial burden imposed by health care expenditure on chronic health conditions such as kidney, heart, liver, and respiratory diseases. However, more investigation is needed to definitively determine the kinds of disabilities most likely to be associated with food insecurity. 

In particular, women with disabilities often experience the triple burden of having a disability, being poor, and being female. Women with disabilities are more likely to be poorly educated, unable to find work, poor, and excluded than men with disabilities; they also face more barriers to accessing services, information, and basic rights [38,44]. It is regrettable that the research on disability and food insecurity has not, to date, significantly explored this interaction between gender and disability-related effects. 

Our research had several limitations. First, KNHANES data on disability registration status, type, and severity are self-reported, such that some inaccuracies are likely. However, Korea has a national registration system for PWD, in which disability types and severity are defined according to specific criteria and the medical diagnosis. Our participants were asked whether they were officially registered on this system as a disabled person, so it is unlikely that their survey responses would have been substantially different from those records. Second, the food security measure, which was designed to identify limitations on food consumption due to financial problems, likely promoted underreporting of HFI due to physical limitations. Some individuals with disabilities who are able to afford food but have limited ability to procure, prepare, or cook it may not be well represented by this measure of food insecurity [20]. Therefore, further research is needed to assess food insecurity in relation to physical and cognitive limitations with respect to securing and preparing adequate food. Finally, the most up-to-date survey data could not be analyzed due to the limited availability of such data on disability status. 

Despite these limitations, this study is informative and valuable in that it attempted to identify food security disparities according to disability status, which is an underrepresented area of food security policy and practice. Also, the study provided detailed information on rates of HFI according to the sociodemographic characteristics of households with disabled members and to the types and severity of disability. Moreover, it identified disability-related factors associated with HFI that were not adequately addressed in previous studies. Finally, the study has important implications for public health and nutrition policy.

The higher prevalence of HFI consistently observed in households with PWD suggests that disability may lead to major nutritional and health disparities at the individual and household levels. It also indicates that current food assistance benefits and disability allowances are not sufficiently flexible and effective in reducing food insecurity among households affected by disabilities. 

Therefore, it is important to develop comprehensive policies to accurately identify vulnerable individuals and families and to better understand and respond to their needs. For instance, nutrition and/or food assistance policies, nutrition education, and dietary counseling targeting disabled women, children with disabilities, and specific disability types could enable more PWDs to access benefits. Disability not only limits economic access to food but also causes substantial difficulties in diet-related activities such as meal planning, transportation to grocery stores, shopping, and meal preparation; thus, additional and more flexible benefits for these households may be required.

To address this important public health issue, we propose (1) greater flexibility in food assistance benefits for PWD, (2) differentiation among types of disabled beneficiaries, and (3) provision of integrated services to meet the complex needs of PWD and the households in which they live. In addition, we hope that future research on food security for PWD will be evaluated in terms of nutritional quality of food consumption and will focus on the nutritional and consequent health disparities between PWD and nondisabled populations.

## 5. Conclusions

In this study, food insecurity is a significant problem among households with PWD. Certain sociodemographic and disability characteristics of PWD were significantly and independently associated with HFI even after further adjustment for socioeconomic variables that could affect the economic status of the household. The results suggest that disability may be an independent risk factor that has a unique effect on food insecurity. It also means that the mechanisms of how disability affects food insecurity should be further explored. Finally, this study improves our understanding of the epidemiology of food insecurity among households with PWDs and could support public policy and programs aimed at addressing food insecurity.

## Figures and Tables

**Table 1:** Characteristics of households with and without persons with disabilities.

	Households with PWD	Households without PWD	*p* Value
	*n* (%)	*n* (%)	
Total Households (*n* = 2690)	384 (14.3)	2306 (85.7)	
Characteristics of Household Head			
Gender			0.02
Male	223 (58.1)	1484 (64.4)	
Female	161 (41.9)	822 (35.7)	
Age, Years			
Mean ± SD	60.7 ± 13.0	53.0 ± 14.7	<0.001
<40	29 (7.6)	478 (20.7)	<0.001
40–49	45 (11.7)	549 (23.8)	
50–59	98 (25.5)	487 (21.1)	
60–69	89 (23.2)	400 (17.4)	
≥70	123 (32.0)	392 (17.0)	
Marital Status			0.81
Living with Spouse	283 (73.7)	1686 (73.1)	
Not Living with Spouse	101 (26.3)	620 (26.9)	
Educational Level			<0.001
Middle School Graduate or Less	209 (54.4)	717 (31.1)	
High School Graduate	84 (21.9)	658 (28.5)	
College Graduate or More	51 (13.3)	668 (29.0)	
Unknown	44 (10.4)	263 (11.4)	
Type of Occupation			<0.001
White-Collar Jobs	43 (11.2)	510 (22.1)	
Blue-Collar Jobs	131 (34.1)	894 (38.8)	
Unemployed	170 (44.3)	635 (27.5)	
Unknown	40 (10.4)	267 (11.6)	
Characteristics of Household			
Household Size (No. of Household Members)			
Mean ± SD	2.7 ± 1.2	2.8 ± 1.2	0.13
1–2 Persons	206 (53.7)	1033 (44.8)	0.003
3 Persons	83 (21.6)	537 (23.3)	
≥4 Persons	95 (24.7)	736 (31.9)	
Family Type			<0.001
One-Person Household	55 (14.3)	411 (17.8)	
Married Couple	116 (30.5)	441 (19.1)	
Married Couple and Unmarried Children	93 (24.2)	928 (40.2)	
Single Parent and Unmarried Children	30 (7.8)	243 (10.5)	
Three-Generational Household	43 (11.2)	123 (5.3)	
Other Household Type	47 (12.2)	160 (6.9)	
Presence of Children Aged ≤18 Years			<0.001
No	287 (74.7)	1395 (60.5)	
Yes	97 (25.3)	911 (39.5)	
No. of Economically Active Household Members			<0.001
None	154 (40.1)	650 (28.2)	
1 Person	136 (35.4)	961 (41.7)	
≥2 Persons	94 (24.5)	695 (30.1)	
Household Income (Quartiles)			<0.001
First Quartile (Poorest)	154 (40.3)	531 (23.1)	
Second Quartile	86 (22.5)	612 (26.7)	
Third Quartile	79 (20.7)	555 (24.2)	
Fourth Quartile (Richest)	63 (16.5)	597 (26.0)	
Place of Residence			0.10
Metropolitan Cities	173 (45.1)	995 (43.2)	
Cities	119 (31.0)	837 (36.3)	
Rural Areas	92 (24.0)	474 (20.6)	
Disability-Related Characteristics			
No. of PWD			
1 Person	356 (92.7)		
≥2 Persons	28 (7.3)		
No. of Working-Age Adults with Disabilities (Aged 19–64 Years)			
None	185 (48.2)		
1 Person	184 (47.9)		
≥2 Persons	15 (3.9)		
No. of Economically Active PWD			
None	247 (64.3)		
1 Person	132 (34.4)		
≥2 Person	5 (1.3)		
Relationships of PWD to Their Families			
Male Head of Household	132 (34.4)		
Female Head of Household	72 (18.8)		
Spouse of Household Head	87 (22.7)		
Son/Daughter of Household Head	33 (8.6)		
Other Relative	34 (8.9)		
Multiple (e.g., Household Head and Spouse, Household Head and Others, and Spouse and Others)	26 (6.8)		
Type of Disability			
Physical Disability	240 (62.5)		
Visual Disability	36 (9.4)		
Auditory or Linguistic Disability	44 (11.5)		
Mental Disability	26 (6.8)		
Internal Organ Disability	18 (4.7)		
Unknown	20 (5.2)		
Severity of Disability			
Severe (Grades 1–3)	119 (31.0)		
Mild (Grades 4–6)	240 (62.5)		
Unknown	25 (6.5)		

Abbreviations: PWD, persons with disabilities; SD, standard deviation.

**Table 2:** Food access and food security status in households with and without persons with disabilities.

	Households with PWD	Households without PWD	*p* Value
	*n* (%)	*n* (%)	
Participation in Food Assistance Programs during the Last 12 Months			<0.001
No	333 (86.7)	2158 (93.6)	
Yes	51 (13.3)	148 (6.4)	
Experiences of Food Insufficiency during the Last 12 Months			0.001
Food-Sufficient	344 (89.6)	2168 (94.0)	
Food-Insufficient	40 (10.4)	138 (6.0)	
Percentage of Households Reporting Insecurity for Every Indicator of HFI during the Last 12 Months			
The Food that We Bought just did not Last and We did not Have Money to Get More	42 (10.9)	182 (7.9)	0.04
Worried whether Food would Run Out before We Got Money to Buy More	100 (26.0)	371 (16.1)	<0.001
Could not Afford to Eat Balanced Meals	97 (25.3)	401 (17.4)	<0.001
Cut Size of Meals or Skipped Meals	12 (3.1)	54 (2.3)	0.36
Ate Less than Felt should	34 (8.9)	133 (5.8)	0.02
Hungry but did not Eat Because could not Afford Food	14 (3.7)	50 (2.2)	0.08
Lost Weight Because Not Enough Money for Food	15 (3.9)	35 (1.5)	0.001
Did not Eat for Whole Day because Not Enough Money for Food	1 (0.3)	12 (0.5)	0.50
Household’s Food Security Status			0.004
Food-Secure	330 (85.9)	2105 (91.3)	
Mildly Food-Insecure	44 (11.5)	162 (7.0)	
Moderately or Severely Food-Insecure	10 (2.6)	39 (1.7)	

Abbreviations: PWD, persons with disabilities; HFI, household food insecurity.

**Table 3:** Results of multivariate logistic regression models predicting household food insecurity.

	Model 1 ^a^	*p* Value	Model 2 ^b^	*p* Value	Model 3 ^c^	*p* Value
	Adjusted OR (95% CI) ^d^		Adjusted OR (95% CI) ^d^		Adjusted OR (95% CI) ^d^	
**Households without PWD (Reference Group)**	1.00		1.00			
**Households with PWD**						
All Households with PWD	1.64 (1.16–2.31)	0.005	1.41 (0.99–2.00)	0.06	1.30 (0.91–1.87)	0.15
Number of PWD (Per Person)	1.51 (1.13–2.02)	0.006	1.35 (1.01–1.81)	0.05	1.25 (0.92–1.69)	0.15
Household Head’s Gender						
Male-Headed Households with PWD	1.20 (0.72–2.01)	0.48	1.11 (0.65–1.87)	0.57	0.96 (0.56–1.65)	0.32
Female-Headed Households with PWD	2.11 (1.37–3.25)	0.007	1.68 (1.08–2.59)	0.06	1.63 (1.04–2.55)	0.04
Household Head’s Disability Status						
Households with A Household Head without A Disability	1.31 (0.73–2.35)	0.74	1.20 (0.66–2.18)	0.85	1.11 (0.61–2.05)	0.79
Households with A Male Head with A Disability	1.30 (0.69–2.45)	0.75	1.06 (0.55–2.03)	0.51	0.90 (0.47–1.76)	0.31
Households with A Female Head with A Disability	2.37 (1.40–4.02)	0.02	1.99 (1.16–3.39)	0.05	1.98 (1.14–3.43)	0.03
Containing Working-Age adults with Disabilities (Aged 19–64 Years)						
No	1.16 (0.71–1.90)	0.34	1.04 (0.63–1.71)	0.31	0.98 (0.59–1.63)	0.30
Yes	2.23 (1.44–3.44)	0.003	1.84 (1.18–2.88)	0.02	1.70 (1.07–2.70)	0.04
Containing Economically Active PWD						
No	1.98 (1.35–2.91)	0.005	1.63 (1.09–2.44)	0.05	1.53 (1.01–2.31)	0.06
Yes	0.96 (0.49–1.91)	0.28	0.93 (0.46–1.86)	0.38	0.84 (0.41–1.72)	0.30
Type of Disability						
Physical Disability	1.39 (0.90–2.15)	0.40	1.19 (0.77–1.84)	0.32	1.16 (0.74–1.82)	0.52
Visual Disability	2.15 (0.84–5.52)	0.62	1.87 (0.72–4.85)	0.66	1.38 (0.53–3.57)	0.99
Auditory or Linguistic Disability	0.44 (0.10–1.89)	0.04	0.45 (0.10–1.92)	0.06	0.47 (0.11–2.04)	0.10
Mental Disability	4.19 (1.69–10.36)	0.04	3.87 (1.53–9.78)	0.04	2.81 (1.08–7.28)	0.12
Internal Organ Disability	4.65 (1.54–14.09)	0.05	3.93 (1.20–12.86)	0.08	4.38 (1.21–15.79)	0.05
Severity of Disability						
Severe (Grades 1–3)	2.13 (1.27–3.56)	0.14	1.87 (1.11–3.17)	0.15	1.73 (1.01–2.99)	0.14
Mild (Grades 4–6)	1.39 (0.89–2.17)	0.82	1.19 (0.76–1.86)	0.73	1.13 (0.72–1.78)	0.90

Abbreviations: OR, odds ratio; CI, confidence interval; PWD, person with disabilities. ^a^ Model 1: Adjusted for age, gender and marital status of household head, household size, family type, presence of children aged ≤ 18 years, and place of residence. ^b^ Model 2: Adjusted for educational level of household head and number of economically active household members in addition to the covariates in model 1. ^c^ Model 3: Adjusted for household income in addition to the covariates in model 2. ^d^ Odds ratios are compared with the control group without PWDs.
